# Multimodality deep learning radiomics predicts pathological response after neoadjuvant chemoradiotherapy for esophageal squamous cell carcinoma

**DOI:** 10.1186/s13244-024-01851-0

**Published:** 2024-11-15

**Authors:** Yunsong Liu, Yi Wang, Xinyang Hu, Xin Wang, Liyan Xue, Qingsong Pang, Huan Zhang, Zeliang Ma, Heping Deng, Zhaoyang Yang, Xujie Sun, Yu Men, Feng Ye, Kuo Men, Jianjun Qin, Nan Bi, Jing Zhang, Qifeng Wang, Zhouguang Hui

**Affiliations:** 1 Department of Radiation Oncology, National Cancer Center/National Clinical Research Center for Cancer/Cancer Hospital, Chinese Academy of Medical Sciences and Peking Union Medical College, Beijing, China; 2 Department of Radiation Oncology, Radiation Oncology Key Laboratory of Sichuan Province, Sichuan Clinical Research Center for Cancer, Sichuan Cancer Hospital & Institute, Sichuan Cancer Center, Affiliated Cancer Hospital of University of Electronic Science and Technology of China, Chengdu, China; 3 Department of Radiation Oncology, Tianjin Medical University Cancer Institute and Hospital, National Clinical Research Center for Cancer, Key Laboratory of Cancer Prevention and Therapy, Tianjin’s Clinical Research Center for Cancer, Tianjin, China; 4 Department of Pathology, National Cancer Center/National Clinical Research Center for Cancer/Cancer Hospital, Chinese Academy of Medical Sciences and Peking Union Medical College, Beijing, China; 5 Department of Diagnostic Radiology, Radiation Oncology Key Laboratory of Sichuan Province, Sichuan Clinical Research Center for Cancer, Sichuan Cancer Hospital & Institute, Sichuan Cancer Center, Affiliated Cancer Hospital of University of Electronic Science and Technology of China, Chengdu, China; 6 Department of VIP Medical Services, National Cancer Center/National Clinical Research Center for Cancer/Cancer Hospital, Chinese Academy of Medical Sciences and Peking Union Medical College, Beijing, China; 7 Department of Diagnostic Radiology, National Cancer Center/National Clinical Research Center for Cancer/Cancer Hospital, Chinese Academy of Medical Sciences and Peking Union Medical College, Beijing, China; 8 Department of Thoracic Surgery, National Cancer Center/National Clinical Research Center for Cancer/Cancer Hospital, Chinese Academy of Medical Sciences and Peking Union Medical College, Beijing, China; 9 Laboratory for Biomechanics and Mechanobiology of Ministry of Education, Beijing Advanced Innovation Center for Biomedical Engineering, School of Engineering Medicine, School of Biological Science and Medical Engineering, Beihang University, Beijing, China

**Keywords:** Esophageal neoplasms, Multimodal imaging, Deep learning, Treatment outcome, Neoadjuvant chemoradiotherapy

## Abstract

**Objectives:**

This study aimed to develop and validate a deep-learning radiomics model using CT, T2, and DWI images for predicting pathological complete response (pCR) in patients with esophageal squamous cell carcinoma (ESCC) undergoing neoadjuvant chemoradiotherapy (nCRT).

**Materials and methods:**

Patients with ESCC undergoing nCRT followed by surgery were retrospectively enrolled from three institutions and divided into training and testing cohorts. Both traditional and deep-learning radiomics features were extracted from pre-treatment CT, T2, and DWI. Multiple radiomics models were developed, both single modality and integrated, using machine learning algorithms. The models’ performance was assessed using receiver operating characteristic curve analysis, with the area under the curve (AUC) as a primary metric, alongside sensitivity and specificity from the cut-off analysis.

**Results:**

The study involved 151 patients, among whom 63 achieved pCR. The training cohort consisted of 89 patients from Institution 1 (median age 62, 73 males) and the testing cohort included 52 patients from Institution 2 (median age 62, 41 males), and 10 in a clinical trial from Institution 3 (median age 69, 9 males). The integrated model, combining traditional and deep learning radiomics features from CT, T2, and DWI, demonstrated the best performance with an AUC of 0.868 (95% CI: 0.766–0.959), sensitivity of 88% (95% CI: 73.9–100), and specificity of 78.4% (95% CI: 63.6–90.2) in the testing cohort. This model outperformed single-modality models and the clinical model.

**Conclusion:**

A multimodality deep learning radiomics model, utilizing CT, T2, and DWI images, was developed and validated for accurately predicting pCR of ESCC following nCRT.

**Critical relevance statement:**

Our research demonstrates the satisfactory predictive value of multimodality deep learning radiomics for the response of nCRT in ESCC and provides a potentially helpful tool for personalized treatment including organ preservation strategy.

**Key Points:**

- After neoadjuvant chemoradiotherapy, patients with ESCC have pCR rates of about 40%.
- The multimodality deep learning radiomics model, could predict pCR after nCRT with high accuracy.
- The multimodality radiomics can be helpful in personalized treatment of esophageal cancer.

**Graphical Abstract:**

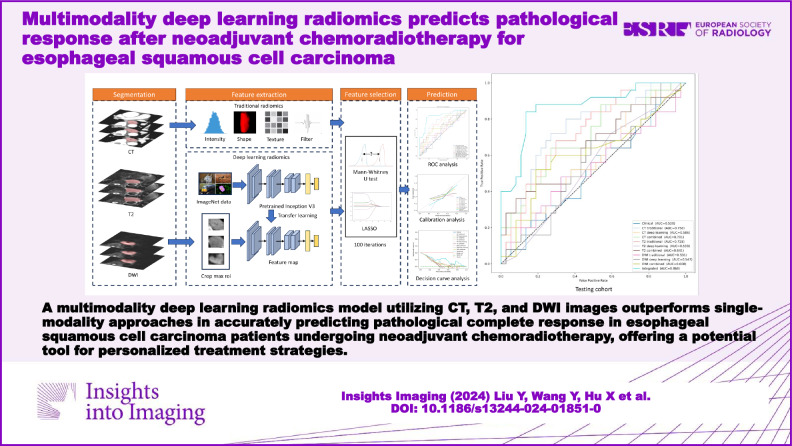

## Introduction

The advancement in managing resectable esophageal squamous cell carcinoma (ESCC) is significantly marked by the evolution of neoadjuvant chemoradiotherapy (nCRT) followed by surgery [1]. This approach has led to a pathological complete response (pCR) rate of 43.2–49%, and has demonstrated a 23% absolute improvement in overall survival (OS) at 10 years when compared to surgery alone [2–4]. However, treatment responses varied among patients, with 36.4% of patients developing recurrence or death at 5 years [5]. Notably, a significant correlation exists between pCR and both OS and disease-free survival [6]. Moreover, pCR status can influence subsequent treatment strategies, as patients who do not achieve pCR may benefit from additional immunotherapy to improve outcomes [7]. However, the confirmation of pCR is only possible through esophagectomy. Accurate prediction of pCR carries substantial implications for patient outcomes. In cases where pCR is likely, OS is comparable following a ‘wait and see’ strategy or standard esophagectomy, potentially circumventing the morbidity associated with surgery [8]. Conversely, for patients unlikely to achieve pCR with standard nCRT, proceeding to surgery remains essential to achieve optimal outcomes.

Previous research has highlighted the distinct value of CT, T2-weighted imaging (T2WI), diffusion-weighted imaging (DWI), and PET/CT modalities in evaluating pCR following nCRT for esophageal cancer [9, 10]. Yang et al [11] developed radiomics models based on pre-treatment CT images to predict pCR, attaining an area under the curve (AUC) of 0.79 in their test dataset. Similarly, Li et al [12] used ADC values from DWI acquired after the completion of nCRT, achieving an accuracy of 71.4% in pCR prediction. In a study by Vollenbrock et al [13], qualitative assessment of T2WI and DWI demonstrated moderate diagnostic performance in predicting pCR, with AUCs ranging from 0.65–0.68 for T2WI and improving to 0.70–0.71 when combined with DWI. However, these studies have faced limitations due to the lack of external validation cohorts from independent institutions and the need for enhanced performance to fulfill clinical requirements. Despite these advances, a concerted effort to amalgamate these modalities into a comprehensive, unified predictive model—which could capitalize on the unique strengths of each modality to further boost performance—remains a significant unexplored avenue.

Radiomics, renowned for its ability to detect intricate patterns and features in imaging data that are often beyond the perceptual capacity of the human eye and conventional analysis methods, is increasingly recognized for its efficacy in predicting treatment responses [14]. The application of radiomics, particularly using pre-treatment CT images in esophageal cancer treated with nCRT, has been extensively researched and shown to hold considerable value [15]. Deep learning, especially via convolutional neural networks (CNNs), has emerged as a markedly superior approach compared to traditional radiomics [16, 17]. Its prowess in autonomously analyzing and interpreting complex, high-dimensional medical imaging data has proven to yield a more in-depth understanding of tumor characteristics. The integration of traditional and deep-learning radiomics offers a comprehensive assessment of tumors. For instance, Wang et al [18] combined these two methods and achieved an impressive AUC of 0.89–0.90 in their testing sets for predicting occult lymph node metastasis in laryngeal squamous cell carcinoma, surpassing the performance of either deep learning or traditional radiomics alone. This combined approach may have also the potential to predict pCR in ESCC.

In light of these insights, our study proposed a novel multimodality deep learning radiomics approach, amalgamating the distinct strengths of CT, T2, and DWI scans, by combining both traditional and deep learning radiomics. We aimed to develop and validate a synergistic model to provide an accurate, comprehensive prediction of pCR in patients undergoing nCRT for ESCC.

## Methods

The study received approval from the institutional review board. Due to the retrospective design, the requirement for informed consent was waived. The study adhered to the checklist for evaluation of radiomics research guidelines [19] to ensure comprehensive and transparent reporting.

### Patients

This multi-cohort study enrolled patients from three institutions, comprising two retrospective cohorts and a retrospective analysis of a cohort from a prospective clinical trial. The inclusion criteria were as follows: (a) pathologically confirmed ESCC, (b) receipt of nCRT and curative resection, and (c) availability of pre-treatment contrast-enhanced CT and MR data, including DWI and T2WI. The exclusion criteria included: (a) insufficient image quality due to obvious artifacts and (b) incomplete clinical and pathological data. Patients for the retrospective cohorts were recruited between September 2014 and September 2023 from institution 1 and between December 2017 and August 2021 from institution 2. The clinical trial cohort comprised patients enrolled in the KEYSTONE-002 trial at institution 3 until November 2023. The KEYSTONE-002 trial is an ongoing phase III randomized controlled trial registered at ClinicalTrials. gov (NCT04807673), with the main inclusion criteria being (a) pathologically confirmed ESCC, (b) R0 resectable thoracic esophageal cancer, cT1-3N1-2M0, cT2-3N0M0, (c) age 18-75 years old, and (d) Eastern Cooperative Oncology Group Performance Status (ECOG-PS) 0–1. All patients underwent curative resection, which involved transthoracic esophagectomy with two-field or three-field lymphadenectomy. Pre-treatment imaging, including contrast-enhanced CT and MR, was performed within two weeks prior to the initiation of nCRT. Patients from institution 1 were allocated to the training cohort, and those from institutions 2 and 3 to the testing cohort.

### Clinical and pathological data collection

Clinical data including age, sex, ECOG-PS, tumor location, tumor length, TNM stage (AJCC 8th edition), chemotherapy regimen, radiotherapy technology, and radiotherapy dose were collected. All patients underwent endoscopy, with most cases using it to determine tumor location and length by measuring the distance from the incisors, categorized according to the AJCC 8th edition. In rare instances where endoscopy could not pass, CT was used for assessing tumor location and length. Clinical staging was assessed using contrast-enhanced CT and MR. Endoscopic ultrasound was utilized except in rare cases where the probe could not pass. ^18^F-FDG PET-CT was performed in 35 patients (23.2%) who had suspected metastatic disease not clearly identified on CT or MR imaging. Clinical staging was determined from initial imaging reports prepared by experienced radiologists and endoscopists at each institution. The staging procedures were consistent across all institutions, including the cohort at Institution 3. Pathologic tumor regression grade was determined postoperatively using the method described by Mandard et al [20]. The therapeutic response was categorized into five grades. Mandard grade 1 with negative lymph node metastasis was categorized as pCR, and others as non-pCR, based on the surgical pathologic examination report.

### CT and MR technique

All patients underwent pre-nCRT contrast-enhanced CT and MR scans. Portal venous phase CT images were collected, with scanning parameter details provided in eTable 1. MR scanning parameter details are shown in eTable 2.

### Tumor segmentation

Tumor regions were manually segmented on multiple contiguous axial slices of contrast-enhanced CT, T2WI, and DWI images to cover the entire tumor volume, by two radiologists, each with five years of experience in thoracic imaging interpretation. The segmentation results were reviewed by two senior experts with 15 years and 25 years of experience, respectively. For interobserver reproducibility analysis, an additional radiologist with 6 years of experience independently segmented tumors in 20 randomly selected patients from the training cohort. The manual segmentation process is illustrated on eFig. 1.

### Radiomic analysis

#### Feature extraction

N4 bias field correction was utilized for MR sequences to address image inhomogeneity [21]. Images were resampled isotropically to a voxel dimension of 1 × 1 × 1 to standardize voxel spacing. To mitigate noise and discretize intensities, the Hounsfield units of CT images were adjusted to the standard abdominal window, setting the window center at 50 and window width at 350. *Z*-score normalization was implemented before extracting traditional features. A total of 1652 features were extracted from both original and filtered images (eTable 3). For deep learning, the Inception-V3 network [22], pre-trained on ImageNet data, was employed. The largest tumor images from each patient were cropped for feature extraction, with grayscale values normalized within the range [−1, 1] using min–max transformation. The images were then resized to 299 × 299 using the nearest interpolation. The network’s last fully connected layer was removed, and the average pooling layer of the feature maps was used to extract 2048 deep-learning features.

#### Feature selection

The feature selection process in this study was conducted within the training cohort. Only features with an intraclass correlation coefficient (ICC) greater than 0.8 were retained. The training cohort was divided into an internal training set and an internal validation set in a 4:1 ratio, a procedure replicated across 100 iterations. In each iteration, the internal training set underwent analysis using the Mann–Whitney *U*-test and least absolute shrinkage and selection operator (LASSO) with 5-fold cross-validation. These methods were employed to generate a feature set for model construction. Ten algorithms were used to build classifiers: logistic regression (LR), support vector machine (SVM), K-nearest neighbors (KNN), decision tree, random forest, extra trees, XGBoost, multi-layer perceptron (MLP), Naive Bayes, and light gradient boosting machine (LightGBM). The performance of these classifiers was tested on the internal validation set. The best-performing classifier and its feature set from each iteration were recorded. Features were ranked based on their frequency of selection. The top two features for each imaging modality (CT and MRI) and feature extraction method (traditional radiomics and deep learning) were selected to build single modality models using the ten algorithms. Then for each imaging modality, a combined model based on both traditional and deep-learning radiomics features was built. To enhance the model’s generalizability and reduce overfitting, another round of feature selection and model construction was performed, again over 100 iterations, based on the twelve features selected in the former procedure. This process aimed to select the top four features for building integrated models with the ten algorithms.

#### Model construction

Five-fold cross-validation was performed with selected feature sets for each modality with ten algorithms (LR, SVM, KNN, Decision Tree, Random Forest, Extra Trees, XGBoost, MLP, Naïve Bayes, and LightGBM). The evaluation of algorithmic performance was based on the calculation of the mean AUC, which served as the principal metric for algorithm selection. Subsequently, the algorithm that exhibited superior performance, as indicated by the highest mean AUC, was selected for the development of the dedicated machine learning model using the training cohort corresponding to each specific modality.

#### Assessment of model performance

**Fig. 1:**
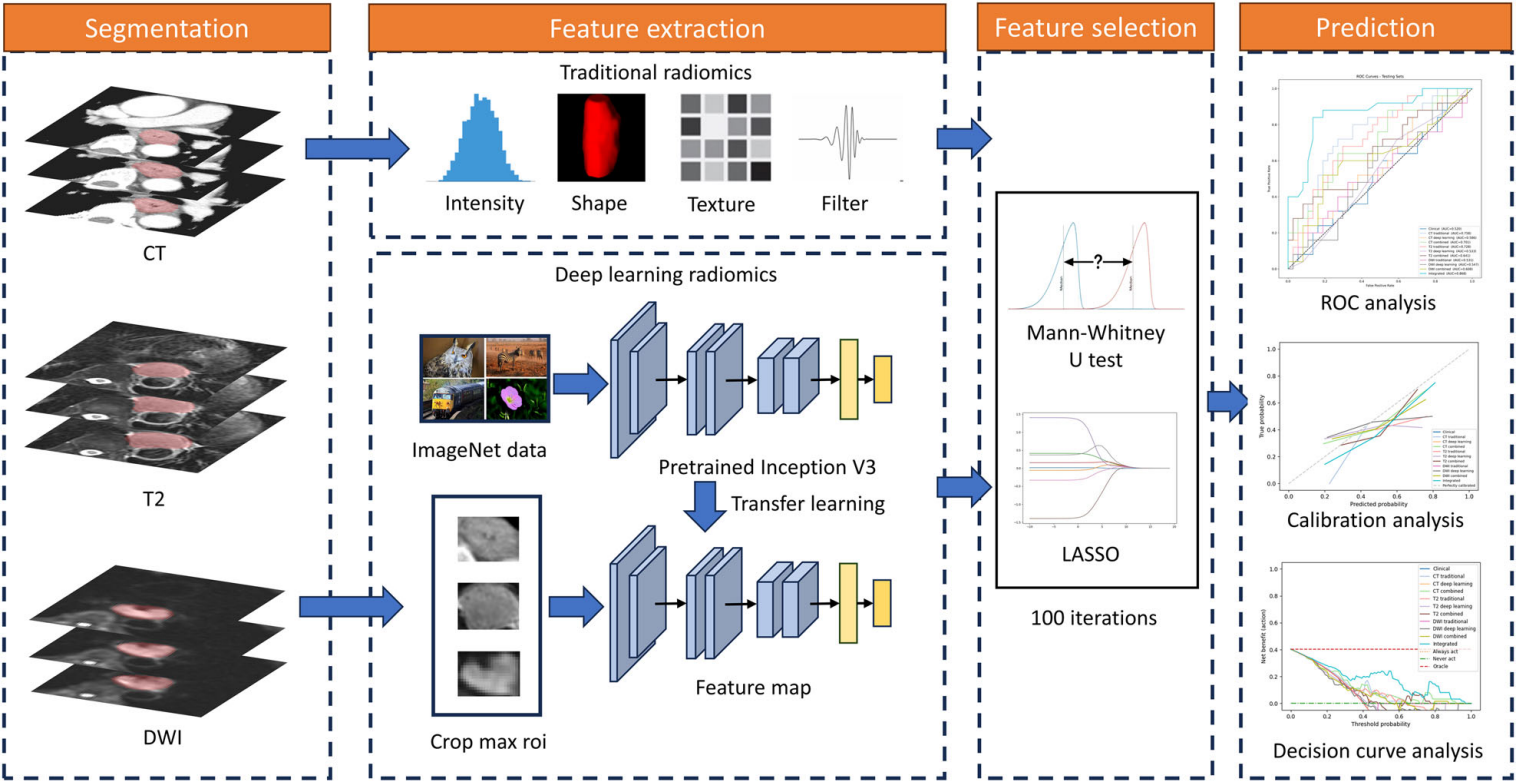
Workflow of the radiomics analysis

Receiver operating characteristic curve analysis and AUC were used to evaluate the models, with the 95% confidence intervals (CIs) being generated through bootstrap resampling, performed 1000 times. The models with ten algorithms were tested independently in the testing cohort and the best was kept to represent the model’s performance. To determine the most effective threshold for the radiomics score, the Youden index was maximized in the training cohort, and these optimal cutoff values were subsequently applied to the testing cohort, and the sensitivity, specificity, positive predictive value (PPV), and negative predictive value (NPV) were calculated. The comparison of AUC values among different models was performed using the DeLong method. Calibration curves and decision curve analysis (DCA) were employed to further assess the models’ performance and their clinical utility. The workflow of the radiomics analysis is depicted in Fig. 1.

### Statistical analysis

Statistical analysis in this study was executed using R version 4.1.2 and Python 3.9. Categorical variables were assessed using Fisher’s exact test, while continuous variables were analyzed with the Mann–Whitney *U*-test. A two-sided *p*-value of less than 0.05 was set for statistical significance.

## Results

### Patients

**Fig. 2:**
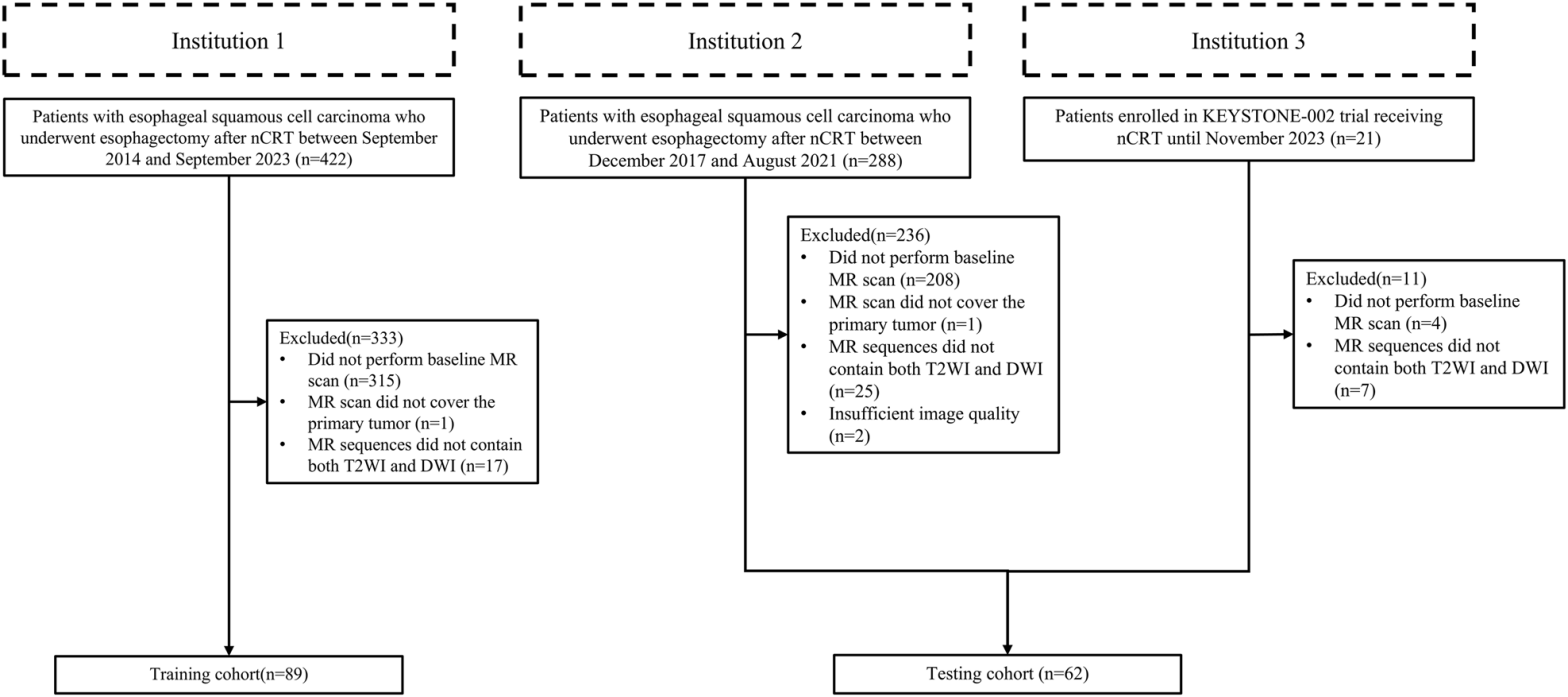
Flowchart diagram shows the patient selection process from three institutions

In this study, out of 731 screened patients, 580 were excluded, resulting in a selection of 151 patients (Fig. 2). The training cohort comprised 89 patients (median age 62, interquartile range (IQR): 57–68, 73 males) from Institution 1. Additionally, the testing cohort comprised 62 patients (median age 62, IQR: 56–68, 50 males), including 52 patients from Institution 2 (median age 62, IQR: 55–67, 41 males) and 10 patients from Institution 3 (median age 69, IQR: 61–71, 9 males). The proportions of patients achieving pCR were 42.7% (38 patients) in the training cohort 40.3% (25 patients) in the testing cohort (36.5% (19 patients) in Institution 2, and 60.0% (6 patients) in Institution 3). Most characteristics were well-balanced between the training and testing cohorts. However, patients in the testing cohort exhibited a better performance status (*p* = 0.002) and a higher utilization of platinum and paclitaxel-based chemotherapy regimens (*p* = 0.015) compared to the training cohort. There were significant differences in radiation technology (*p* < 0.001) between the two cohorts, and the simultaneous integrated boost radiation was exclusively administered in the training cohort. The detailed clinical characteristics of these groups are summarized in Table 1 and eTable 4. It was noted that no clinical factors showed a significant correlation with pCR in both training and testing cohorts.

**Table 1:** Patients’ characteristics

Characteristic	Training cohort				Testing cohort				*p*-value
	All, (*N* = 89)	pCR, (*N* = 38)	Non-pCR, (*N* = 51)	*p*-value	All, (*N* = 62)	pCR, (*N* = 25)	Non-pCR, (*N* = 37)	*p*-value	
Age	62 (57, 68)	64 (58, 68)	62 (57, 68)	0.832	62 (56, 68)	62 (57, 68)	62 (55, 68)	0.897	0.797
Sex				0.097				0.198	0.835
Male	73 (82.0)	28 (73.7)	45 (88.2)		50 (80.6)	18. (72.0)	32 (86.5)		
Female	16 (18.0)	10 (26.3)	6 (11.8)		12 (19.4)	7 (28.0)	5 (13.5)		
ECOG				0.281				0.165	0.002
0	36 (40.4)	18 (47.4)	18 (35.3)		42 (67.7)	14 (66.0)	28 (75.7)		
1	53 (59.6)	20 (52.6)	33 (64.7)		20 (32.3)	11 (44.0)	9 (24.3)		
Tumor location				0.176				0.592	0.652
Upper thoracic	10 (11.2)	7 (18.4)	3 (5.9)		8 (12.9)	3 (12.0)	5 (13.5)		
Middle thoracic	31 (34.8)	11 (28.9)	20 (39.2)		25 (40.3)	12 (48.0)	13 (35.1)		
Lower thoracic	48 (53.9)	20 (52.6)	28 (54.9)		29 (46.8)	10 (40.0)	19 (51.4)		
Tumor length	5.0 (4.0, 7.0)	5.0 (4.0, 6.0)	5.0 (4.0, 7.0)	0.696	6.0 (4.0, 7.0)	6.0 (4.0, 7.0)	5.0 (5.0, 7.0)	0.749	0.679
cT				0.533				0.515	0.974
1	1 (1.1)	0 (0.0)	1 (2.0)		0 (0.0)	0 (0.0)	0 (0.0)		
2	5 (5.6)	2 (5.3)	3 (5.9)		3 (4.8)	2 (8.0)	1 (2.7)		
3	63 (70.8)	25 (65.8)	38 (74.5)		46 (74.2)	19 (76.0)	27 (73.0)		
4	20 (22.5)	11 (28.9)	9 (17.6)		13 (21.0)	4 (16.0)	9 (24.3)		
cN				0.950				0.750	0.299
0	6 (6.7)	3 (7.9)	3 (5.9)		4 (6.5)	2 (8.0)	2 (5.4)		
1	27 (30.3)	11 (28.9)	16 (31.4)		21 (33.9)	10 (40.0)	11 (29.7)		
2	39 (43.8)	16 (42.1)	23 (45.1)		32 (51.6)	11 (44.0)	21 (56.8)		
3	17 (19.1)	8 (21.1)	9 (17.6)		5 (8.1)	2 (8.0)	3 (8.1)		
Chemotherapy regimen				0.801				0.076	0.015
Platinum and paclitaxel	68 (76.4)	30 (78.9)	38 (74.5)		57 (91.9)	25 (100.0)	32 (86.5)		
Others	21 (23.6)	8 (21.1)	13 (25.5)		5 (8.1)	0 (0.0)	5 (13.5)		
Radiation technology				0.739				0.291	< 0.001
IMRT	10 (11.2)	5 (13.2)	5 (9.8)		52 (83.9)	19 (76.0)	33 (89.2)		
VMAT	79 (88.8)	33 (86.8)	46 (90.2)		10 (16.1)	6 (24.0)	4 (10.8)		
Radiation dose	41.4 (37.8, 43.2)	41.4 (37.8, 44)	41.4 (37.8, 41.4)	0.463	40.0 (40.0, 40.0)	40.0 (40.0, 41.4)	40.0 (40.0, 40.0)	0.235	0.117
SIB radiation	56 (62.9)	23 (60.5)	33 (64.7)	0.825	0 (0.0)	0 (0.0)	0 (0.0)		< 0.001

*pCR* pathological complete response, *IQR* interquartile range, *ECOG PS* Eastern Cooperative Oncology Group performance status, *cT* clinical T stage, *cN*, clinical N stage, *IMRT* intensity-modulated radiation therapy, *VMAT* volumetric modulated arc therapy, *SIB* simultaneous integrated boost

^a^ Unless otherwise indicated, data are numbers of patients, and data in parentheses are percentage

^b^ Data are means, with IQRs in parentheses

### Feature selection

Regarding feature selection, after assessing features for satisfactory reproducibility, the study retained 1497, 1606, and 1618 features for traditional radiomics of CT, T2, and DWI, respectively. For deep learning radiomics, 1754, 987, and 2010 features were retained for CT, T2, and DWI, respectively. Following 100 iterations of the *U*-test and LASSO analysis, six traditional radiomics features and six deep learning features were selected (as detailed in eTables 5 and 6). During this process of feature selection, the mean AUCs of single-modality models ranged from 0.484 to 0.750. The best performance was observed in CT traditional radiomics, with a mean AUC of 0.750 using LightGBM, followed by T2 deep learning radiomics, with a mean AUC of 0.706 using KNN or LR. Subsequent analysis, involving an additional 100 iterations focusing on the previously selected 12 features, yielded mean AUCs ranging between 0.750 and 0.839, with the best performance achieved by Extra Trees. This phase culminated in the identification of four pivotal features for the integrated model: one traditional and one deep learning feature from CT, one deep learning feature from T2, and one deep learning feature from DWI (detailed in eTables 7 and 8). Notably, among these, the wavelet-HHL_glszm_LargeAreaHighGrayLevelEmphasis feature from CT stood out for its robustness, being selected in 80 out of the 100 iterations.

### Model construction

The clinical model, designed by incorporating factors deemed crucial by clinical experts—specifically age, sex, cT, and cN, achieved its highest mean AUC of 0.639 with MLP during five-fold cross-validation. For CT images, traditional and deep learning radiomics reached peak mean AUCs of 0.725 (LR) and 0.701 (SVM), respectively. Combining these traditional and deep learning radiomics improved performance, with the highest mean AUC at 0.802 (MLP). For T2 images, traditional and deep learning radiomics recorded the highest mean AUCs of 0.669 (MLP) and 0.763 (KNN), respectively, with the combined model achieving 0.720 (MLP). DWI images showed the highest mean AUCs of 0.633 (MLP) for traditional and 0.750 (SVM) for deep learning radiomics, with the combined model reaching 0.694 (XGboost). The final integrated model showed superior performance than other models for all algorithms except for Naïve Bayes, with the highest mean AUC of 0.835 using Extra Trees (eTable 9).

### Model evaluation

**Fig. 3:**
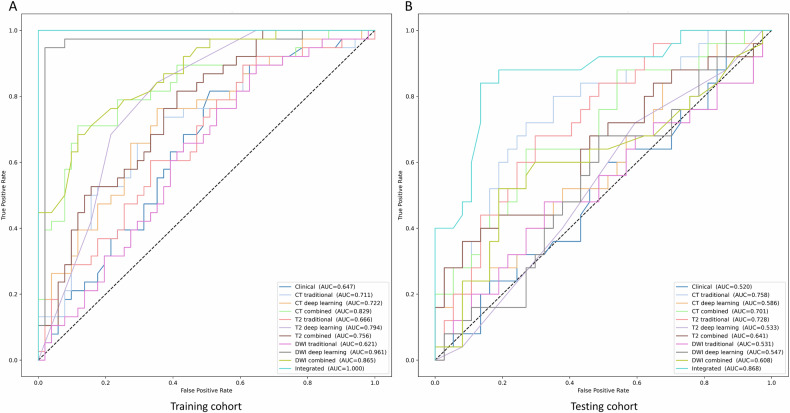
Performances of different models. **A** Performances of different models in the training cohort; **B** Performances of different models in the testing cohort

**Fig. 4:**
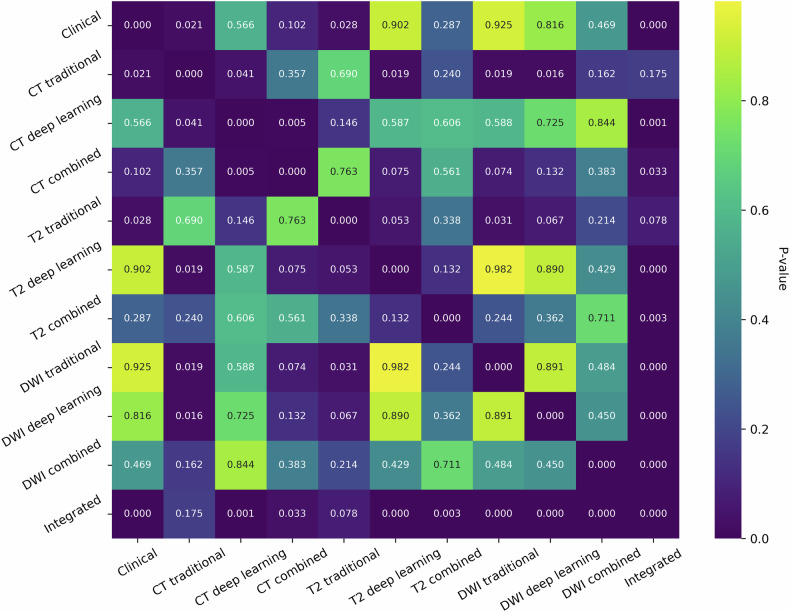
Comparison of different models’ performances in the testing cohort by DeLong test

The clinical model demonstrated an AUC of 0.520 (95% CI: 0.369–0.657) in the testing cohort. For CT images, the traditional radiomics model demonstrated superior performance, achieving an AUC of 0.758 (95% CI: 0.619–0.872). It recorded a sensitivity of 68.0% (95% CI: 50.0–85.7) and specificity of 75.7% (95% CI: 61.5–88.9), alongside a PPV of 65.4% (95% CI: 46.4–83.3) and NPV of 77.8% (95% CI: 64.3–90.6). In T2 images, the traditional radiomics model showed optimal performance with an AUC of 0.728 (95% CI: 0.600–0.852), although applying the training cohort’s cut-off value resulted in a sensitivity of 100% and a specificity of 18% (95% CI: 7.9–31.6). For DWI images, the combined model exhibited the best performance in the testing cohort, with an AUC of 0.604 (95% CI: 0.455–0.763), a sensitivity of 52.0% (95% CI: 32.0–70.8), specificity of 81.1% (95% CI: 66.7–93.6), PPV of 65.0% (95% CI: 41.6–85.7), and NPV of 71.4% (95% CI: 56.4–84.4). The integrated model demonstrated superior performance with an AUC of 0.868 (95% CI: 0.766–0.959), sensitivity of 88% (95% CI: 73.9–100), and specificity of 78.4% (95% CI: 63.6–90.2), along with a PPV of 73.3% (95% CI: 56.5–88.0) and NPV of 90.6% (95% CI: 80.6–100.0). The DeLong test comparing the performance between the training and testing cohorts showed a significant difference (*p* < 0.001). The comprehensive performance of all models is displayed in Table 2 and Fig. 3. The integrated model achieved AUCs of 0.875 and 0.958 in a retrospective set and clinical trial set of the testing cohort, respectively (eFigure 2). The integrated model outperformed all other models in the study, demonstrating the highest effectiveness. This superiority is marked by significant differences compared to all other models (*p* < 0.05 in the DeLong test) except for CT traditional radiomics model and T2 traditional radiomics model in performance metrics, as detailed in Fig. 4. DCA indicated that the integrated model offered the most clinical benefit (eFigure 3), and calibration analysis confirmed its satisfactory calibration (eFigure 4).

**Table 2:** Performances of models^a^

	AUC	Sensitivity, (%)	Specificity, (%)	PPV, (%)	NPV, (%)
Clinical model					
Training cohort	0.647 (0.532–0.762)	81.6 (68.6–93.3)	49.0 (35.1–63.0)	54.4 (41.8–67.9)	78.1 (62.1–91.4)
Testing cohort	0.520 (0.369–0.657)	60.0 (41.4–78.6)	45.9 (30.3–63.2)	42.9 (27.0–60.0)	63.0 (44.8–80.8)
CT traditional radiomics model					
Training cohort	0.711 (0.596–0.813)	73.7 (59.5–86.1)	62.7 (49.1–75.0)	59.6 (45.1–72.9)	76.2 (62.5–87.8)
Testing cohort	0.758 (0.619–0.872)	68.0 (50.0–85.7)	75.7 (61.5–88.9)	65.4 (46.4–83.3)	77.8 (64.3–90.6)
CT deep learning radiomics model					
Training cohort	0.722 (0.608–0.826)	76.3 (62.8–89.5)	64.7 (50.9–78.0)	61.7 (47.7–75.7)	78.6 (65.8–90.6)
Testing cohort	0.586 (0.439–0.727)	52.0 (31.8–72.0)	59.5 (42.9–75.6)	46.4 (28.6–64.3)	64.7 (47.2–80.8)
CT combined model					
Training cohort	0.829 (0.728–0.915)	71.1 (55.9–85.1)	88.2 (78.9–96.2)	81.8 (66.7–93.6)	80.4 (69.4–90.5)
Testing cohort	0.701 (0.561–0.822)	44.0 (24.0–65.0)	78.4 (64.7–91.2)	57.9 (35.0–81.0)	67.4 (54.5–81.0)
T2 traditional radiomics model					
Training cohort	0.666 (0.549–0.766)	89.5 (78.9–97.7)	39.2 (25.5–52.1)	52.3 (39.6–64.3)	83.3 (66.7–96.2)
Testing cohort	0.728 (0.600–0.852)	100.0 (100.0–100.0)	18.9 (7.9–31.6)	45.5 (32.1–59.2)	100.0 (100.0–100.0)
T2 deep learning radiomics model					
Training cohort	0.794 (0.700–0.876)	84.2 (72.4–95.1)	64.7 (51.0–77.3)	64.0 (50.9–77.2)	84.6 (72.5–95.2)
Testing cohort	0.533 (0.397–0.663)	80.0 (63.0–93.8)	27.0 (13.5–41.2)	42.6 (29.2–57.4)	66.7 (41.2–88.9)
T2 combined model					
Training cohort	0.756 (0.658–0.841)	81.6 (68.6–92.9)	58.8 (44.9–71.4)	59.6 (46.2–72.6)	81.1 (67.4–92.9)
Testing cohort	0.641 (0.485–0.723)	84.0 (68.2–96.4)	29.7 (15.4–44.1)	44.7 (30.0–59.2)	73.3 (46.7–94.1)
DWI traditional radiomics model					
Training cohort	0.621 (0.505–0.735)	89.5 (78.1–97.6)	35.3 (21.8–48.1)	50.7 (38.1–62.5)	81.8 (62.5–96.2)
Testing cohort	0.531 (0.384–0.681)	96.0 (87.0–100.0)	2.7 (0.0–8.3)	40.0 (27.9–51.7)	50.0 (0.0–100.0)
DWI deep learning radiomics model					
Training cohort	0.961 (0.903–1.000)	94.7 (86.1–100.0)	98.0 (93.6–100.0)	97.3 (91.1–100.0)	96.2 (90.6–100.0)
Testing cohort	0.547 (0.401–0.685)	16.0 (3.8–31.8)	86.5 (75.0–96.9)	44.4 (10.0–80.0)	60.4 (47.0–73.2)
DWI combined model					
Training cohort	0.865 (0.782–0.931)	68.4 (54.1–83.3)	88.2 (78.2–96.4)	81.2 (66.7–94.1)	78.9 (67.3–88.7)
Testing cohort	0.608 (0.455–0.763)	52.0 (32.0–70.8)	81.1 (66.7–93.6)	65.0 (41.6–85.7)	71.4 (56.4–84.4)
Integrated model					
Training cohort	1.000	100.0 (100.0–100.0)	100.0 (100.0–100.0)	100.0 (100.0–100.0)	100.0 (100.0–100.0)
Testing cohort	0.868 (0.766–0.959)	88.0 (73.9–100.0)	78.4 (63.6–90.2)	73.3 (56.5–88.0)	90.6 (80.6–100.0)

*AUC* area under the curve, *PPV* positive predictive value, *NPV* negative predictive value

^a^ 95% CIs in brackets

## Discussion

The present study introduced an approach to the prediction of pCR in ESCC following nCRT, leveraging a multimodality deep learning radiomics framework. This approach synergized the unique strengths of various imaging modalities—CT, T2, and DWI—along with both traditional and deep learning radiomics, forming a comprehensive and robust predictive model. Our model demonstrated an AUC of 0.868 in a multi-institutional testing cohort, marking it as the first of its kind to employ multimodality deep learning radiomics for predicting pCR in ESCC after nCRT.

Prior studies largely focused on using single-modality imaging for predicting pCR. A meta-analysis reported AUCs ranging from 0.65 to 0.86 for radiomics studies using either PET, CT, or MRI [23]. However, these studies often lacked comprehensive external validation, with only three studies having an external validation set from a single institution, showing AUCs between 0.79 and 0.85 [11, 24, 25]. Our multimodality imaging approach overcomes several limitations of previous research. Single modality models, while valuable, are often unable to fully capture tumor heterogeneity and its response to therapy [26, 27]. In contrast, our integrated model, harnessing CT, T2, and DWI scans, exhibited superior performance compared to single-modality models. This suggests that combining multiple imaging techniques allows for more nuanced tumor analysis and the identification of subtle treatment response indicators that might be overlooked with single-modality imaging.

The integrated model incorporated one traditional radiomics feature and three deep-learning radiomics features. Specifically, the GLSZM feature from CT imaging provided a quantification of gray-level intensity variations, reflecting intratumor heterogeneity [28]. Furthermore, wavelet-transformed features, while complex, offered more intricate insights into tumor heterogeneity. In consistence with our study, the wavelet GLSZM feature of CT was found to be valuable in predicting pCR of rectal cancer after nCRT [29].

The integration of deep learning radiomics into our model is a crucial element of our study. Presently, there is a notable scarcity of research employing deep learning techniques for predicting pCR in ESCC. In this context, Hu et al’s work, which involved the application of a pretrained deep learning model, stands out. They achieved an AUC of 0.805 in predicting pCR, thereby surpassing the results obtained through traditional radiomics methods [25]. Our study, however, takes this a step further by utilizing the pre-trained Inception-V3 network on multimodality imaging, which led to an even higher level of performance. This improvement could be attributed to the synergistic combination of traditional radiomics with deep learning, as indicated by a meta-analysis that the combined model outperformed in 63% of the examined studies, and had comparable performance in 13% [30]. Additionally, our study uniquely leveraged T2 and DWI imaging modalities, known for their superior soft tissue contrast and functional information, which have not been previously explored with deep learning for predicting pCR. Hirata et al [31] used pretreatment histogram-derived ADC from DWI of 58 patients, finding that skewness was the best predictor for pCR, achieving an AUC of 0.86. Traditional radiomics using T2 imaging achieved an AUC of 0.83 in the internal testing set in predicting the response of neoadjuvant chemotherapy of ESCC [32]. Notably, all these studies lacked multicenter external testing sets. In contrast, our model not only exhibited better performance but also demonstrated generalized applicability, suggesting that deep learning may more effectively harness the information provided by MR sequences.

Despite its strengths, our study recognizes several limitations. Firstly, our data collection was retrospective in nature, potentially introducing biases associated with patient selection and variations in historical treatments. Secondly, an AUC of 1 in the training cohort and the results of the DeLong test suggest potential overfitting, predominantly due to the relatively small sample size. However, 5-fold cross-validation demonstrated satisfactory performance, aligning well with the external testing set and indicating good generalizability. To mitigate potential overfitting and improve model robustness, we plan to expand the sample size in future studies. Thirdly, the process of manually segmenting tumor regions in imaging studies is both time-intensive and laborious. To address this challenge, we are actively pursuing research in auto-segmentation techniques. Additionally, MRI is not recommended as a standard tool for staging esophageal cancer yet, which could restrict the reproducibility and external validation of our model in institutions where MRI is not commonly used. Furthermore, PET/CT is recommended by guidelines for staging in candidates for esophagectomy [33], but it was performed only when clinically indicated in our study. The lack of consistent PET/CT utilization could have influenced the accuracy of tumor staging and the evaluation of tumor heterogeneity, thereby affecting the generalization of our results. Lastly, our study did not delve into the biological mechanisms underlying the predictive model. To further enhance our understanding and the robustness of our model, future research will focus on prospective validation and integrate genomic and transcriptomic data, thereby enriching the model’s predictive capability with a more comprehensive biological context.

In conclusion, this study developed and validated an accurate multimodality deep learning radiomics model in predicting the pCR of ESCC following nCRT. It underscored the potential of combining multimodality imaging and deep learning in this research field, and also contributed to the advancement of personalized care of esophageal cancer.

## Supplementary information

ELECTRONIC SUPPLEMENTARY MATERIAL

## Data Availability

All data and materials are available through a reasonable request to the corresponding author.

## Abbreviations

AUC: Area under the curve
CI: Confidence interval
DCA: Decision curve analysis
DWI: Diffusion weighted imaging
ESCC: Esophageal squamous cell carcinoma
IQR: Interquartile range
KNN: K-nearest neighbors
LASSO: Least absolute shrinkage and selection operator
LightGBM: Light gradient boosting machine
LR: Logistic regression
MLP: Multi-layer perceptron
nCRT: Neoadjuvant chemoradiotherapy
NPV: Negative predictive value
OS: Overall survival
pCR: Pathological complete response
PPV: Positive predictive value
SVM: Support vector machine
T2WI: T2-weighted imaging
