# Supplementary material for: Multimodality deep learning radiomics predicts pathological response after neoadjuvant chemoradiotherapy for esophageal squamous cell carcinoma
Source: Insights Imaging. 2024 Nov 15;15:277. doi: 10.1186/s13244-024-01851-0 (PMC11568088; doi:10.1186/s13244-024-01851-0)
Supplement: Supplementary file 1 — ELECTRONIC SUPPLEMENTARY MATERIAL [file 13244_2024_1851_MOESM1_ESM.pdf]

Multimodality deep learning radiomics predicts pathological response after neoadjuvant chemoradiotherapy for esophageal squamous cell carcinoma

ELECTRONIC SUPPLEMENTARY MATERIAL

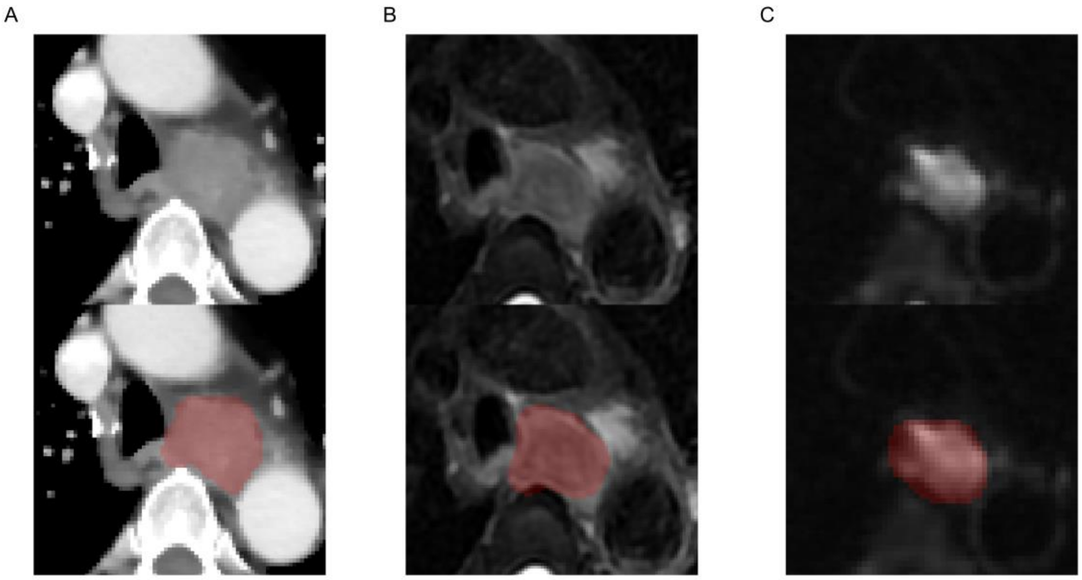

eFigure 1. Manual segmentation of regions of interest (ROI) on three imaging modalities in one patient. Segmentations are shown on axial slices of (A) contrast-enhanced CT, (B) T2-weighted imaging, and (C) diffusion-weighted imaging. The ROI is highlighted in red, clearly demarcating the tumor boundaries.

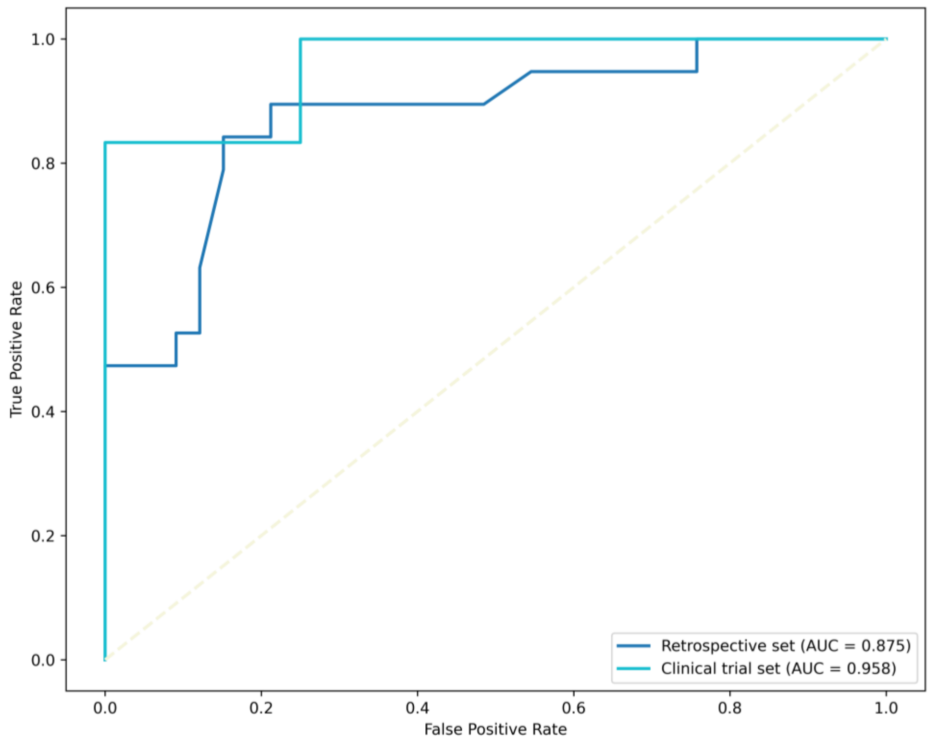

eFigure 2. Performances of the integrated model in retrospective set and clinical trial set of the testing cohort. AUC, area under the curve.

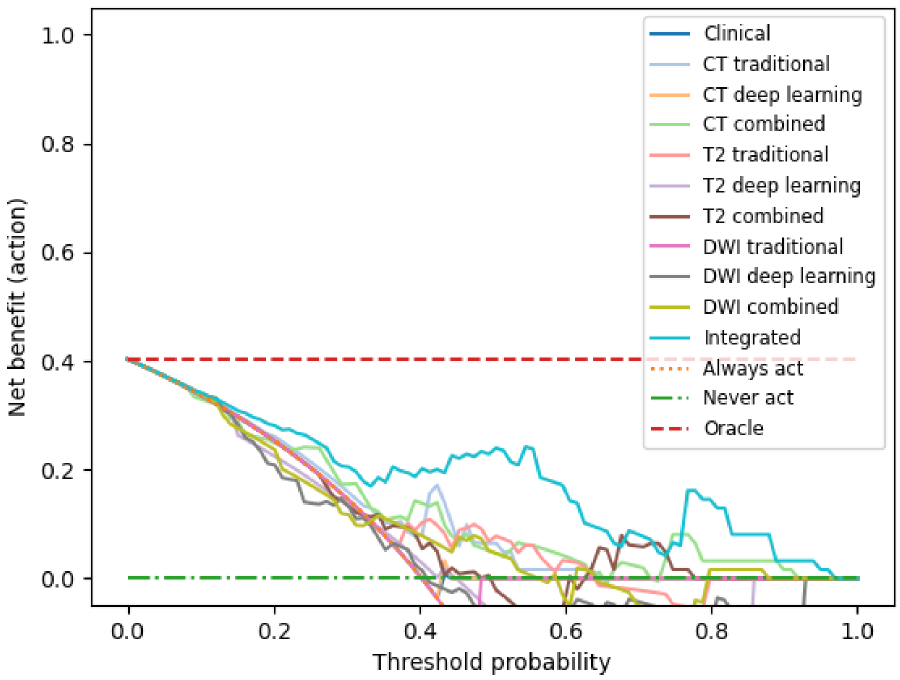

eFigure 3. Decision curve analysis of models in the testing cohort.

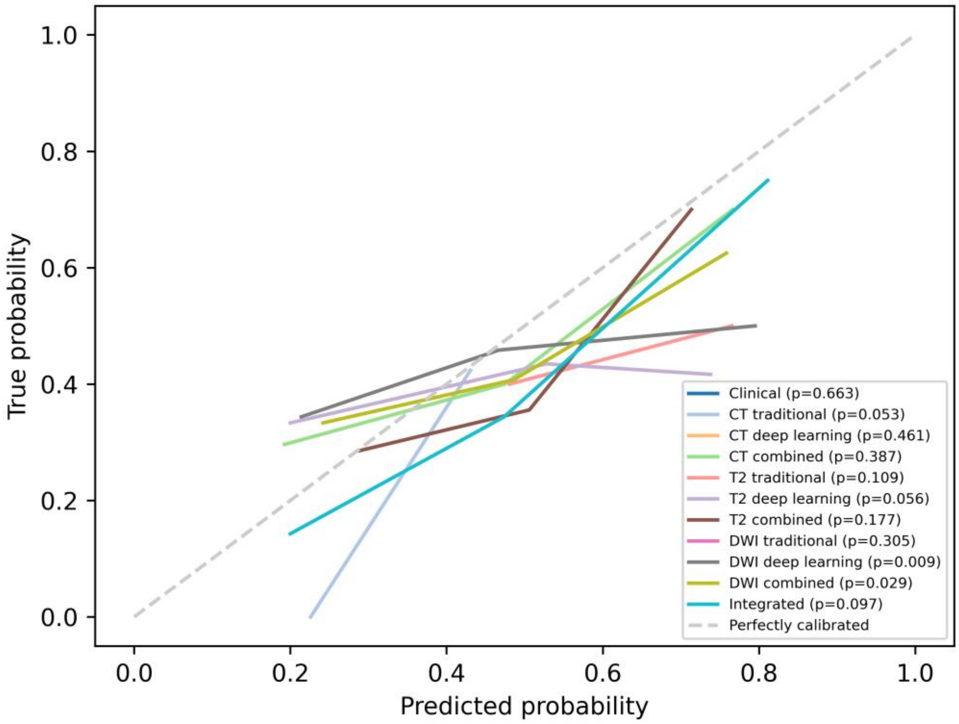

eFigure 4. Calibration curves of models in the testing cohort.

eTable1. Scanning parameters of CT

Institution	Scanner	Tube voltage(kV)	Tube current(mA)	Matrix	Slice thickness
1	Siemens Definition AS	120	560	512*512	5
	Philips Brilliance	120	220	512*512	5
2	Philips Brilliance	120	375	512*512	5
3	GE Revolution EVO	120	250	512*512	5
	GE Optima CT680 Expert	120	250	512*512	5
	Siemens Definition AS	120	400	512*512	5

eTable2. Scanning parameters of T2 and DWI

Institution	Scanner	Sequence	Repetition time (ms)	Echo time (ms)	Echo train length (ms)	Flip angle (°)	Matrix	Slice thickness
1	GE Discovery MR750w 3.0T	T2	11250	88	32	90/142	512*512	5
		DWI	12000	68	59	90	256*256	5
	GE Discovery MR750 3.0T	T2	18000	99	32	90	512*512	5
		DWI	10000	57	50	90	256*256	5
2	SIEMENS Skyra 3.0T	T2	4500	83	43	110	320*320	5
		DWI	6200	61	51	90	208*256	5
	UIH uMR588 1.5T	T2	4000	84	28	120	432*432	5
		DWI	3800	75	44	90	202*256	5
3	Philips Ingenia 3.0T	T2	7700	95	28	90	448*448	5
		DWI	3000	76	47	90	528*528	5

eTable 3. Extracted traditional radiomics features

Feature types	Number of features (n=1652)
Shape features	14
First-order features	18
NGTDM features	5
GLCM features	22
GLRLM features	16
GLSZM features	16
GLDM features	14
LoG features	273
Wavelet features	728
LBP features	273
Exponential features	91
Square features	91
Logarithm features	91

eTable 4. Patients' characteristics [a]

Characteristic	Training cohort (N=89)	Testing cohort (N=62)	
	Institution 1<br>N = 89	Institution 2<br>N = 52	Institution 3<br>N = 10
pCR	38 (42.7)	19 (36.5)	6 (60.0)
Age	62 (57, 68)	62 (55, 67)	69 (61, 71)
Sex			
Male	73.0 (82.0)	41.0 (78.8)	9.0 (90.0)
Female	16.0 (18.0)	11.0 (21.2)	1.0 (10.0)
ECOG PS			
0	36 (40.4)	42 (80.8)	0 (0.0)
1	53 (59.6)	10.0 (19.2)	10.0 (100.0)
Tumor location			
Upper thoracic	10.0 (11.2)	8.0 (15.4)	0.0 (0.0)
Middle thoracic	31.0 (34.8)	21.0 (40.4)	4.0 (40.0)
Lower thoracic	48.0 (53.9)	23.0 (44.2)	6.0 (60.0)
Tumor length	5.0 (4.0, 7.0)	5.0 (4.0, 7.0)	6.1 (5.3, 7.0)
cT			
1	1 (1.1)	0 (0.0)	0 (0.0)
2	5 (5.6)	3 (5.8)	0 (0.0)
3	63 (70.8)	36 (69.2)	10 (100.0)
4	20 (22.5)	13 (25.0)	0 (0.0)
cN			
0	6 (6.7)	1 (1.9)	3 (30.0)
1	27 (30.3)	19 (36.5)	2 (20.0)
2	39 (43.8)	27 (51.9)	5 (50.0)
3	17 (19.1)	5 (9.6)	0 (0.0)
Chemotherapy regimen			
Platinum and paclitaxel	68 (76.4)	47 (90.4)	10 (100.0)
Others	21 (23.6)	5 (9.6)	0 (0.0)
Radiation technology			
IMRT	10 (11.2)	52 (100.0)	10 (100.0)
VMAT	79 (88.8)	0 (0.0)	0 (0.0)
Radiation dose	41.4 (37.8, 43.2)	40.0 (40.0, 40.0)	41.4 (41.4, 41.4)
SIB radiation	56 (62.9)	0 (0.0)	0 (0.0)

[a]Unless otherwise indicated, data are numbers of patients, and data in parentheses are percentage. [b]Data are means, with IQRs in parentheses. pCR, pathological complete response; IQR, interquartile range; ECOG PS, Eastern Cooperative Oncology Group Performance Status; cT, clinical T stage; cN, clinical N stage; IMRT, Intensity-Modulated Radiation Therapy; VMAT, Volumetric Modulated Arc Therapy; SIB, simultaneous integrated boost.

eTable 5. Performance of different modalities using ten algorithms in 100 iterations

Classifier	CT-traditional		T2-traditional		DWI-traditional		CT-deep learning		T2-deep learning		DWI-deep learning	
	Number of Iterations	Mean AUC (When Best)	Number of Iterations	Mean AUC (When Best)	Number of Iterations	Mean AUC (When Best)	Number of Iterations	Mean AUC (When Best)	Number of Iterations	Mean AUC (When Best)	Number of Iterations	Mean AUC (When Best)
Decision Tree	8	0.597	13	0.555	7	0.621	11	0.615	17	0.585	14	0.595
Extra Trees	8	0.677	10	0.627	7	0.681	4	0.672	6	0.606	9	0.599
K-Nearest Neighbor	8	0.595	13	0.609	19	0.639	14	0.657	10	0.706	15	0.625
LightGBM	17	0.750	7	0.527	12	0.636	22	0.650	9	0.675	17	0.647
Logistic Regression	8	0.700	7	0.591	8	0.663	4	0.631	8	0.706	3	0.692
Multilayer Perceptron	5	0.713	4	0.484	10	0.620	0	-	20	0.688	5	0.603
Naïve Bayes	21	0.687	13	0.581	4	0.616	8	0.653	13	0.650	4	0.569
Random Forest	6	0.715	6	0.605	8	0.653	16	0.674	5	0.629	10	0.689
Support Vector Machine	8	0.669	20	0.599	14	0.585	9	0.621	6	0.633	10	0.620
XGBoost	11	0.685	7	0.573	11	0.671	12	0.642	6	0.667	13	0.617

eTable 6. Frequencies of selections of each feature for different modalities

**CT-traditional**		**T2-traditional**		**DWI-traditional**		**CT-deep learning**		**T2-deep learning**		**DWI-deep learning**	
**Feature**	**Frequency**	**Feature**	**Frequency**	**Feature**	**Frequency**	**Feature**	**Frequency**	**Feature**	**Frequency**	**Feature**	**Frequency**
wavelet-HHL_glszm_LargeAreaHighGrayLevelEmphasis	74	lbp-3D-m2_firstorder_Maximum	21	wavelet-LLL_glcm_ClusterShade	37	DL1337	85	DL194	94	DL1118	40
logarithm_glcm_ClusterProminence	23	wavelet-HHH_glcm_ClusterProminence	16	original_shape_MajorAxisLength	18	DL680	70	DL1298	86	DL1830	17
wavelet-LHL_glszm_LargeAreaHighGrayLevelEmphasis	15	square_gldm_LargeDependenceLowGrayLevelEmphasis	14	wavelet-LLL_firstorder_Skewness	18	DL1280	69	DL1165	84	DL1915	16
wavelet-HLL_glszm_LargeAreaHighGrayLevelEmphasis	15	logarithm_firstorder_10Percentile	12	original_firstorder_Skewness	17	DL689	58	DL382	54	DL1795	14
logarithm_ngtdm_Complexity	7	wavelet-HHL_firstorder_Median	12	log-sigma-2-0-mm-3D_firstorder_Kurtosis	13	DL923	55	DL2002	51	DL1629	11

eTable 7. Performance of the former selected 12 features using ten algorithms

Classifier	Number of Iterations	Mean AUC (When Best)
Decision Tree	5	0.750
Extra Trees	6	0.839
0K-Nearest Neighbor	9	0.788
LightGBM	23	0.773
Logistic Regression	4	0.756
Multilayer Perceptron	7	0.759
Naïve Bayes	4	0.794
Random Forest	17	0.779
Support Vector Machine	9	0.781
XGBoost	16	0.792

eTable 8. Frequencies of selections of the former selected 12 features

Feature	Frequency
CT_wavelet-HHL_glszm_LargeAreaHighGrayLevelEmphasis	80
T2_DL194	20
CT_DL1337	20
DWI_DL1118	17
T2_DL1298	15
CT_DL680	14
DWI_DL1830	9
T2_lbp-3D-m2_firstorder_Maximum	7
DWI_wavelet-LLL_glcm_ClusterShade	5
T2_wavelet-HHH_glcm_ClusterProminence	3
CT_logarithm_glcm_ClusterProminence	2
DWI_original_shape_MajorAxisLength	2

eTable 9. Performances in 5-fold cross validation

Classifier	Mean AUC										
	**Clinical**	**CT traditional radiomics**	**CT deep learning**	**CT combined**	**T2 traditional radiomics**	**T2 deep learning**	**T2 combined**	**DWI traditional**	**DWI deep learning**	**DWI combined**	**Integrated**
Decision Tree	0.551	0.600	0.648	0.708	0.539	0.709	0.581	0.502	0.578	0.598	0.734
Extra Trees	0.442	0.626	0.562	0.719	0.464	0.729	0.646	0.447	0.697	0.608	0.835
K-Nearest Neighbor	0.555	0.650	0.651	0.729	0.584	0.763	0.627	0.493	0.708	0.655	0.758
LightGBM	0.506	0.601	0.655	0.739	0.585	0.712	0.665	0.525	0.660	0.656	0.822
Logistic Regression	0.580	0.725	0.690	0.789	0.631	0.734	0.708	0.572	0.622	0.624	0.778
Multilayer Perceptron	0.639	0.713	0.701	0.802	0.669	0.745	0.720	0.633	0.722	0.686	0.812
Naïve Bayes	0.594	0.666	0.677	0.666	0.591	0.709	0.699	0.524	0.638	0.524	0.666
Random Forest	0.416	0.587	0.599	0.727	0.495	0.685	0.618	0.548	0.679	0.674	0.824
Support Vector Machine	0.507	0.630	0.701	0.786	0.627	0.731	0.714	0.582	0.750	0.669	0.818
XGBoost	0.504	0.637	0.642	0.692	0.593	0.720	0.703	0.524	0.715	0.694	0.762
